# *Galleria mellonella* Infection Model Identifies Both High and Low Lethality of *Clostridium perfringens* Toxigenic Strains and Their Response to Antimicrobials

**DOI:** 10.3389/fmicb.2019.01281

**Published:** 2019-07-03

**Authors:** Sammy Kay, Joseph Edwards, Joseph Brown, Ronald Dixon

**Affiliations:** ^1^School of Life Sciences, Joseph Banks Laboratories, University of Lincoln, Lincoln, United Kingdom; ^2^Arden Biotechnology, Lincoln, United Kingdom

**Keywords:** alternative model, insect model, host-pathogen interactions, infectivity, greater wax moth

## Abstract

Research progress into mechanisms of the anaerobe *Clostridium perfringens* and associated diseases has been frustrated by the lack of reliable infection models. Wax moth larvae (*Galleria mellonella*) have emerged as a viable alternative to other models of infection since they are economic, survive at 37°C and require no specialist equipment. This study aims to establish to what extent *G. mellonella* larvae can be used to study the virulence of *C. perfringens* strains and its suitability for studying novel treatment strategies by an improved time-lapse approach to data collection. Mortality and morbidity rates of larvae challenged with 10^5^ CFU of *C. perfringens* isolates from various sources were observed over 72 h and dose response data obtained. Phenoloxidase enzyme activity was investigated as a marker for immune response and tissue burden assessed by histopathological techniques. Results demonstrate that *C. perfringens* is pathogenic toward *G. mellonella* although potency varies dramatically between *C. perfringens* isolates and the reference strain ATCC 13124 was shown to be avirulent. Infection with *C. perfringens* strains activated the melanisation pathway resulting in melanin deposition but no increase in enzyme activity was observed. Efficacy of antibiotic therapy (penicillin G, bacitracin, neomycin, and tetracycline) administered parenterally to some extent correlates with that of *in vitro* analysis. The findings suggest *G. mellonella* might be a useful *in vivo* model of infection and convenient as a pre-screening assay for virulence of *C. perfringens* strains or as a simple, cheap and rapid *in vivo* assay in the first stage development of novel therapeutics against anaerobes.

**HIGHLIGHTS:**

– Potential novel *in vivo* model for the study of *Clostridium perfringens* infection.
– Novel time-lapse approach to data collection.
– First report of the pathogenicity of *C. perfringens* toward *G. mellonella*.
– First report of the efficacy of antibiotic therapy in response to *C. perfringens* infection in *G. mellonella*.

## Introduction

Insect models have been shown to be helpful in our understanding of the virulence of bacterial pathogens in humans (Tsai et al., 2016). Insects share some similarities with mammalian processes and possess a basic innate immune system (Ramarao et al., 2012). Alternative model organisms such as the zebra fish (*Danio rerio*), silkworm (*Bombyx mori*), tobacco hornworm (*Manduca sexta*), fruit fly (*Drosophila melanogaster*), and nematodes (*Caenorhabditis elegans*) (Browne et al., 2013) are not associated with the same ethical considerations as in the use of higher vertebrates (Peterson et al., 2008). The larval stage of the Greater Wax moth (*Galleria mellonella*) has emerged as an insect model of particular value since it survives at 37°C, which is essential for demonstration of specific microbial virulence factors (Smoot et al., 2001).

The larvae are inexpensive to obtain and easy to maintain using basic equipment (Ramarao et al., 2012). The model does not require ethical approval and coupled with fast reproduction time, allows for a high-throughput of experiments compared with mammalian systems. Whilst *G. mellonella* has not evolved an adaptive immune response, they possess a semi-complex cellular and humoral innate immunity. This innate system, in insects, shares remarkable similarities to that of mammals (Jander et al., 2000). The cellular immune response in the larvae consists of haemocytes which play a role in phagocytosis, encapsulation and clotting as an antimicrobial response (Tojo et al., 2000). The humoral response consists of various antimicrobial peptides, opsonins, extracellular nucleic acid traps and the phenol-oxidase pathway as described recently in detail by Tsai et al. (2016).

Development of infection models with *G. mellonella* larval hosts has involved a diverse range of microbes including: *Cryptococcus neoformans* (Mylonakis et al., 2005), *Burkholderia cepacia* (Seed and Dennis, 2009), *Yersinia pseudotuberculosis* (Champion et al., 2009) *Acinetobacter baumannii* (Peleg et al., 2009), *Campylobacter jejuni* (Senior et al., 2011), *Candida albicans* (Brennan et al., 2002), *Legionella pneumophilia*, (Harding et al., 2013), *Pseudomonas aeruginosa* (Beeton et al., 2015), *Mycobacterium fortuitum* (Entwistle and Coote, 2018), and *Vibrio parahaemolyticus* (Wagley et al., 2017). In the present study, we have investigated the pathogenicity of *C. perfringens* in *G. mellonella* larvae. *C. perfringens* is a ubiquitous Gram-positive, spore-forming, anaerobic bacilli, classically characterized by four major extracellular toxins: alpha (*cpa)*, beta (*cpb)*, epsilon (*etx*), and iota (*iA*). Recently necrotic enteritis-like B toxin (*netB*) and enterotoxin (*cpe*) has been added to the classification system (Table 1; Rood et al., 2018). The bacteria produce a plethora of non-typing toxins including haemolysin, perfringolysin O (*pfoA*), collagenase (*colA*) (Songer, 1996), beta2 toxin (*cpb2*) (Van-Asten et al., 2010), and binary enterotoxin A and B (*becA* and *becB*) (Yonogi et al., 2014) with differing and elusive roles in the bacterial mode of infectivity.

**Table 1:** Toxin type classification system for *C. perfringens.*

Type	Alpha	Beta	Epsilon	lota	Entero-toxin	*netB^∗^*
A	**+**	**-**	**-**	**-**	**-**	**-**
B	**+**	**+**	**+**	**-**	**-**	**-**
C	**+**	**+**	**-**		**+/-**	**-**
D	**+**	**-**	**+**	**-**	**+/-**	**-**
E	**+**	**-**	**-**	**+**	**+/-**	**-**
F	**+**	**-**	**-**	**-**	**+**	**-**
G	**+**	**-**	**-**	**-**	**-**	**+**

*Isolates are characterized by the production of one, or more, of the now six major toxins: alpha, beta, epsilon, iota, ^∗^necrotic enteritis like B and enterotoxin. Alpha toxin is produced by all types of *C. perfringens* (Rood et al., 2018).*

*Clostridium perfringens* is one of the leading food borne pathogens in the United Kingdom and worldwide. It is implicated in 80–95% of reported gas gangrene cases (Titball, 2005) and causes large economic losses in the agricultural industry (Wade et al., 2015). Research into this important bacterial pathogen has been slowed by the lack of standardized disease models for assessing virulence and *in vivo* susceptibility to antimicrobial drugs. The *G. mellonella* larvae model of infection is developing as an alternative screening method for economically progressing novel antimicrobial compounds prior to mammalian studies.

To date, no detailed reports of *C. perfringens* infection models with *G. mellonella* have been published. It should be noted, however, that Champion et al. (2016) highlights an unpublished report suggesting alpha and enterotoxin from *C. perfringens* lack toxicity against the larvae. The aim of this study was to establish the extent of *G. mellonella* susceptibility to *C. perfringens* infection as a basis for further virulence studies. This present study investigates the susceptibility to 15 distinct *C. perfringens* isolates from United Kingdom environmental sources including a standard reference strain and explores the response of the larvae to traditional antibiotic therapy. In addition, we introduce a novel imaging capture and time-lapse methodology designed to increase accuracy whilst easily monitoring larval disease progression.

## Materials and Methods

### Bacterial Strains and Preparation of Inoculum

*Clostridium perfringens* was sourced from our culture collection and isolates selected based on toxin profiles (Table 2; Sim et al., 2015). Cultures were stored in 30% glycerol (Thermo Fisher Scientific, United Kingdom), 70% brain heart infusion (BHI) media (Oxoid, United Kingdom) at -80°C and cultured on BHI agar (Oxoid, United Kingdom). Liquid cultures were grown to mid-log phase, in BHI or thioglycollate broth (FTG) (Oxoid, United Kingdom) and incubated at 37°C for 5 h. Bacterial inocula were adjusted by absorbance (OD_620_) and CFU/mL determined by traditional serial dilution and plating on TSC agar (Oxoid, United Kingdom). Cells in adjusted cultures were pelleted by centrifuging at 3170 ×*g* at room temperature for 2 min (Heraeus Megafuge 8) and washed once with 0.1% peptone water (Oxoid, United Kingdom). Dilutions were drawn into a 1 mL syringe fitted with a 30 g sterile needle (BD plastipak).

**Table 2:** *Clostridium perfringens* toxin profiles of strains and isolates used within this study.

Isolate	Source	*cpa*	*cpb*	*etx*	*iA*	*cpe*	*netb*	*cpb2*	*pfoA*	*tpeL*	*colA*	*becA*	*becB*	*cna*	Type
JBFR014	Free Range Chicken	**+**	**-**	**-**	**-**	**-**	**-**	**-**	**-**	**-**	**-**	**-**	**-**	**-**	A
ATCC 13124	Type Strain	**+**	**-**	**-**	**-**	**-**	**-**	**-**	**+**	**-**	**+**	**-**	**-**	**-**	A
LPCP001	Sheep	**+**	**-**	**-**	**-**	**-**	**-**	**-**	**+**	**-**	**+**	**-**	**-**	**-**	A
LPCP023	Sheep	**+**	**-**	**-**	**-**	**-**	**-**	**-**	**+**	**-**	**+**	**-**	**-**	**-**	A
LPCP042	Sheep	**+**	**-**	**-**	**-**	**-**	**-**	**-**	**+**	**-**	**+**	**-**	**-**	**-**	A
EDCB283	Retail Chicken	**+**	**-**	**-**	**-**	**-**	**+**	**+**	**+**	**-**	**+**	**-**	**-**	**-**	G
EDCB093	Retail Chicken	**+**	**-**	**-**	**-**	**-**	**-**	**-**	**+**	**+**	**+**	**-**	**-**	**-**	A
JBRC013	Broiler	**+**	**-**	**-**	**-**	**-**	**+**	**-**	**-**	**-**	**+**	**+**	**-**	**-**	G
JBCNJ58	Broiler	**+**	**-**	**-**	**-**	**-**	**+**	**-**	**+**	**-**	**+**	**-**	**-**	**-**	G
JBCNCR03	Broiler	**+**	**-**	**-**	**-**	**-**	**+**	**-**	**+**	**+**	**+**	**-**	**-**	**-**	G
JBCNC055	Broiler	**+**	**-**	**-**	**-**	**-**	**-**	**+**	**+**	**-**	**+**	**-**	**-**	**-**	A
JBCND021	Broiler	**+**	**-**	**-**	**-**	**-**	**+**	**+**	**+**	**-**	**+**	**-**	**-**	**-**	G
JBCNI050	Broiler	**+**	**-**	**-**	**-**	**-**	**-**	**-**	**-**	**-**	**-**	**-**	**-**	**-**	A
JBCND053	Broiler	**+**	**-**	**-**	**-**	**-**	**-**	**-**	**+**	**-**	**+**	**-**	**-**	**-**	A
JBCNJ055	Broiler	**+**	**-**	**-**	**-**	**-**	**-**	**+**	**+**	**-**	**+**	**-**	**-**	**-**	A

### *Galleria mellonella* Challenge Assay

**FIGURE 1:**
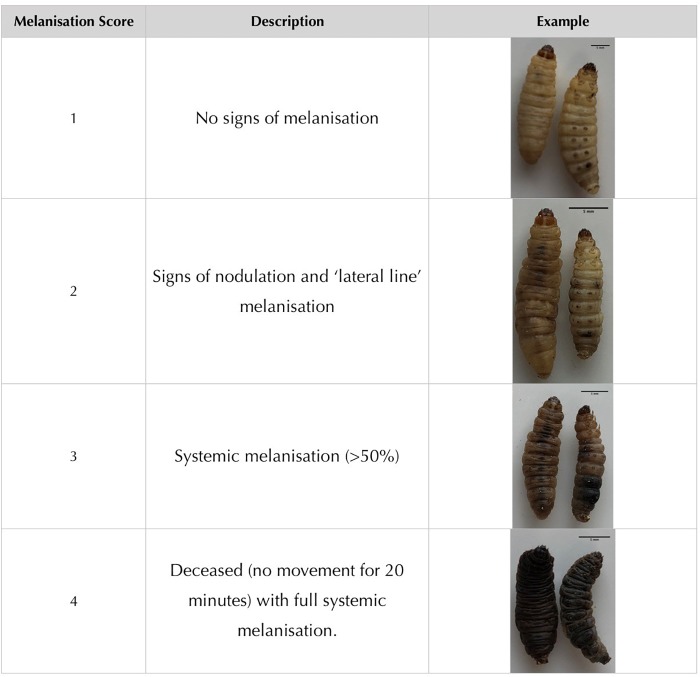
Melanisation scoring system adapted from Senior et al. (2011).

**FIGURE 2:**
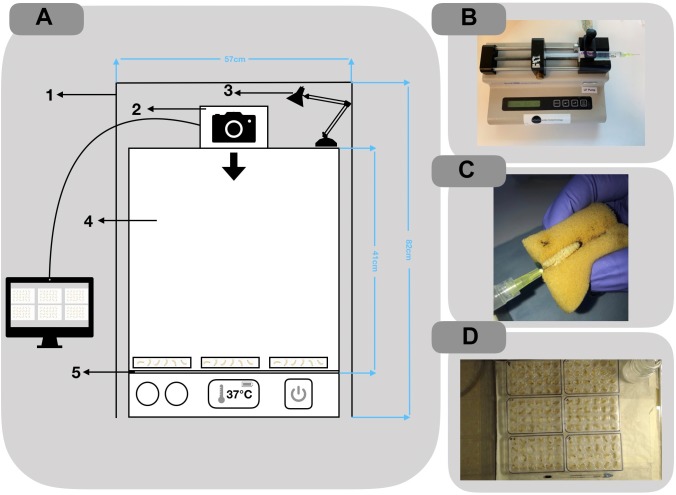
**(A)** Schematic of the imaging setup used to record time-lapse photography of larvae 1: Black felt shroud to reduce the effects of natural light on quality of images; 2: Camera: Logitek C100 5 mp; 3: table lamp for consistent lighting; 4: perspex incubator: Stuart Scientific SI140; 5: Greaseproof paper for better contrast in images. **(B)** Automated syringe pump setup used for inoculating larva. **(C)** Injections were administered through the rear pro-leg. **(D)** Example of image output from SkyStudio Pro time-lapse software.

Larvae were purchased from Live Foods United Kingdom (Rooks Bridge, United Kingdom). Upon arrival, they were stored in wood shavings, at room temperature, in the dark. To avoid sampling biases, larvae with any signs of melanisation or deformity were rejected. Larvae were weighed and only larvae meeting the criteria of 250 ± 50 mg were utilized. Prior to all injections, larvae were immobilized by standing the 24 micro-well plate (Thermo Fisher Scientific, United Kingdom) on ice for 15 min. An automated syringe pump (KD Scientific, United States) was supplied with the drawn 1 mL syringes and set to administer 1 μL/s for 10 s (Figure 2B). A safety restraint device was used, outlined by Dalton et al. (2017) (Figure 2C). To minimize adverse effects, larvae were injected into the right posterior proleg (Figure 2C). A melanisation scoring system adapted from Senior et al. (2011) is shown in Figure 1 and employed throughout. Results were obtained longitudinally with a novel image capture system (Figure 2A). Briefly, the system was constructed with a benchtop incubator, with transparent sides and top (Stuart Scientific SI140). Images were recorded by a Logitech 15 MP camera coupled with time-lapse software (SkystudioPro) (Figure 2D). For each experiment, images of the larvae were recorded every 10 min for 72 h.

### Pathogenicity Screening

Fifteen *C. perfringens* isolates from poultry, goats and their environments along with ATCC 13124 (Table 2) were prepared for inoculation as described above. Groups of 10 larvae were each challenged with 10 μl of 10^7^ CFU/mL and each experiment included 10 larvae injected with 0.1% peptone water as controls. Larvae were placed in Petri-dishes lined with greaseproof paper to allow for greater contrast in images. All groups were incubated aerobically at 37°C and survival recorded at 72 h p.i. The experiments were repeated in triplicate and an average survival result recorded.

### Dose Dependent Challenge

In dose dependent challenge studies, larvae were prepared by placing one per well in 24 micro-well plastic plates (Thermo Fisher Scientific, United Kingdom). All larvae were treated identically to ensure injection and isolate continuity throughout. ATCC 13124, JBFR014, and JBCNJ055 were investigated by preparing a dilution series of each isolate and larvae were injected with a dilution of 10 μL of washed cultures ranging from approximately 10^3^ to 10^7^ CFU/mL. In addition, 24 larvae were injected with 10 μL of 0.1% peptone water as negative controls. All six plates for each isolate were recorded simultaneously with the image recording system described previously. The larvae were incubated for 72 h at 37°C. Images were taken every 10 min for 72 h. Melanisation scores and mortality were recorded at 0,12, 24, 36, 48, 60, and 72 h post injection. All experiments were repeated in triplicate and average scores recorded.

### Soluble Virulence Factor Trial

*JBCNJ055, EDCB0283, EDCB093*, and *JBCND053* (Selection based on distinct toxin profiles or sources) were used in cell free trials. 100 μl of overnight culture, of each isolate was inoculated in 5 mL of FTG (Oxoid, United Kingdom) and incubated at 37°C for 5 h. The culture was centrifuged at 3170 ×*g* (Heraeus Megafuge 8) for 3 min and 1 mL of the supernatant was transferred to a 1.5 mL microcentrifuge tube (Eppendorf, United Kingdom). The remaining supernatant was removed from the bacterial pellet and discarded. The pellet was resuspended in 3 mL of sterile FTG (Oxoid, United Kingdom) and 1 mL of the resuspended culture transferred to a 1.5 mL microcentrifuge tube. The contents of each microcentrifuge tube were drawn into separate 1 mL sterile syringes (BD Plastipak) and fitted with a 30 g needle. Ten larvae were challenged with 10 uL of each inoculate, from each isolate and were placed in sterile Petri-dishes lined with greaseproof paper (for better contrast in images). The plates were incubated at 37°C for 72 h. Survival percentages were recorded, and each experiment repeated in triplicate.

### Isolate Selection for Further Analysis

*Clostridium perfringens* JBCNJ055 was selected as a typical recent isolate obtained from a broiler chicken with putative necrotic enteritis in the United Kingdom. Both JBFR014 and JBCNJ055 have been studied extensively in our laboratory and are scheduled to be DNA sequenced. ATCC 13124 was used as a standard reference strain. The remaining isolates used in this study currently have no further characterization.

### Haemolymph Extraction

Twelve larvae were challenged with 10 μL of approximately 10^7^ CFU/mL of *C. perfringens* JBCNJ055 washed culture and 12 larvae injected with 10 μL of 0.1% peptone water as controls. All larvae were incubated at 37°C simultaneously for 72 h. At 24 h intervals post challenge, three larvae from each group were placed into separate sterile 15 mL centrifuge tubes and submerged in ice for 15 min to immobilize. Haemolymph was extracted by removing the posterior two segments and bleeding into a sterile pre-chilled 1.5 mL micro centrifuge tube (Eppendorf, United Kingdom). 25 μL of extracted haemolymph was transferred into 75 μL of 50 mM PBS (pH 6.5) (Gibco, United Kingdom) then briefly vortexed and centrifuged to pellet the cells at 11,180 ×*g* for 10 min at 4°C. The supernate was transferred into sterile 1.5 mL tubes. Samples were processed within 15 min of extraction to avoid rapid melanisation.

### Phenoloxidase Activity

Phenoloxidase activity was measured using a microplate enzyme assay (Eleftherianos et al., 2008). Briefly, a reaction mixture containing 115 μL of 50 mM PBS (pH 6.5) and 10 μL haemolymph plasma was prepared. 25 μL of 20 mM 4-methyl catechol (Sigma, United Kingdom) was added as enzyme substrate and 2 μL of 10 mM *Escherichia coli* LPS (Sigma, United Kingdom) was added to controls. Plates were subjected to 1 h of low agitation (25 rpm) at room temperature to activate endogenous pro-phenol-oxidase prior to addition of the substrate. The change in absorbance was read at 490 nm for 1 h at room temperature with a reading taken every 60 s using a plate reader (Fluostar Optima). Each reaction was repeated in triplicate.

### Histopathology of *Galleria* Samples

Groups of three larvae were injected with 10 μL of approximately 10^7^ CFU/mL of *C. perfringens* JBCNJ055 and incubated at 37°C for 72 h. Control groups were injected with 10 μL of 0.1% peptone water. At 36 h post injection, larvae were immobilised by submerging in ice for 15 min. Larvae were injected with 150 μL of 10% neutral buffered formalin (Thermo Fisher Scientific, United Kingdom) (for internal fixation) until turgid and stored in 10% neutral buffered formalin for 36 h prior to routine tissue processing. Larvae were dissected completely along the sagittal plane, and both halves embedded into wax blocks. Tissue sections (5 μm) were cut by routine methods, mounted on glass slides and stained with either H+E, Masson Fontana, or Gram stains. Sections were imaged using light microscope (Zeiss Primostar) and a 1080 p camera (Mitotic).

### MIC Determination

*Clostridium perfringens* isolate JBCNJ055 was sub-cultured in 10 mL of thioglycollate broth (Oxoid, United Kingdom) and incubated for 6 h, aerobically at 37°C. Broth dilution MIC assays were produced in microtiter plates and prepared with ranges from 128 to 0.25 μg/mL of penicillin G, bacitracin, tetracycline and neomycin. MICs were recorded as the lowest concentration of antimicrobial agent that completely inhibits growth turbidity compared with positive controls where antibiotics were omitted. MIC testing was repeated in triplicate on three occasions.

### Antibiotic Therapy

The same “batch” of larvae (LiveFoods, United Kingdom) were employed for each antibiotic therapy experiment. As positive controls, 24 larvae were challenged with 10 μL of approximately 10^7^ JBCNJ055 culture followed by an injection of 10 μL of sterile dH_2_O. A further 24 larvae were injected with 10 μL of the same inoculum with a second 10 μL injection of either penicillin G (2 mg/kg), bacitracin (64 mg/kg), tetracycline (64 mg/kg), or neomycin (2400 mg/kg); concentrations injected are shown in Table 3. Treatment injections were administered within 15 min of the bacterial injection. Dosages were chosen to equate with greater than 10 ×*in vitro* MIC. All larvae were subjected to toxicity testing with the maximum doses prior to therapy trials, no melanisation or death was observed. Finally, 24 larvae were injected with 10 μL 0.1% peptone water as negative controls (Oxoid, United Kingdom). Larvae were incubated simultaneously at 37°C for 72 h. Morbidity and mortality scores were recorded at 0, 12, 24, 36, 48, 60, and 72 h post injection. All experiments were repeated in triplicate and average scores recorded.

**Table 3:** *In vitro* MIC results with associated therapeutic dosage.

Treatment	*In vitro* MIC (μg/mL)	> × 10 (μg /mL)	Antibiotic dosage × 100 *(*μ*g/mL)*	Final larval Dosage (μg/larva)	Larval survival (% ± SD)
+ Control				0	73.61 ± 6.36
- Control				0	100 ± 0
Penicillin G	0.18	5	500	5	68.06 ± 15.77^*^
Bacitracin	1.56	15.6	1600	16	94.45 ± 4.8^*^
Neomycin	60	600	60,000	600	65.28 ± 18.78
Tetracycline	1.56	15.6	1600	16	79.17 ± 14.43

*Survival percentages ± Standard deviation after 72 h of being treated with 10 μL injected doses of 10 ×in vitro MIC against 10μL injection of 10^7^ JBCNJ055. “+” indicates no treatment administered. “-” indicates infection with 0.1% peptone water as trauma controls. ^∗^Significantly different when compared to + control (P > 0.05) (Man–Whitney U-test).*

### Statistical Analysis

The data from each repeat was pooled. Analysis for dose dependent challenges was conducted using Kaplan–Meier survival distributions and tested for statistical significance using the Log rank (Mantel Cox) test (pooled over strata). Mann–Whitney test was used to highlight statistical significance unless stated otherwise. All tests were carried out in SPSS, 24 (IBM).

## Results

### Isolate Pathogenicity

**FIGURE 3:**
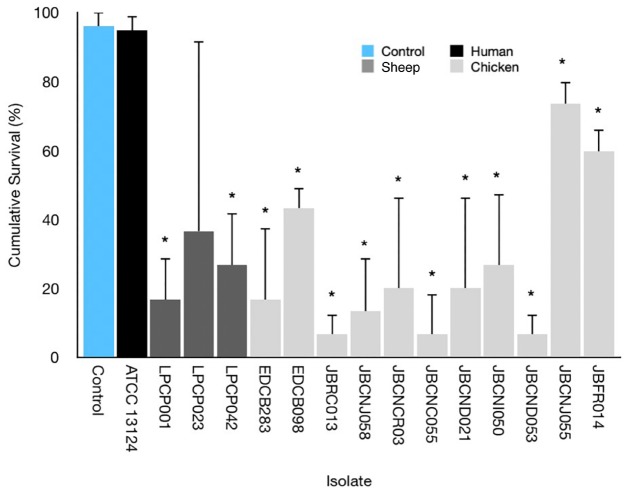
Average survival percentage at 72 h incubation for *G. mellonella* infected with 10 μL of 10^7^ CFU/mL of respective isolates. Error bars indicate standard error. Results indicate a variance in lethality of *C. perfringens* infection toward *G. mellonella*. ^∗^ = Mann–Whitney *U*-test comparing larval survival to uninfected control.

Figure 3 highlights the variability seen in pathogenicity against *G. mellonella* with different isolates of *C. perfringens*. The isolates exhibit a varying degree of pathogenicity with cumulative survival from 5 to 100%.

**FIGURE 4:**
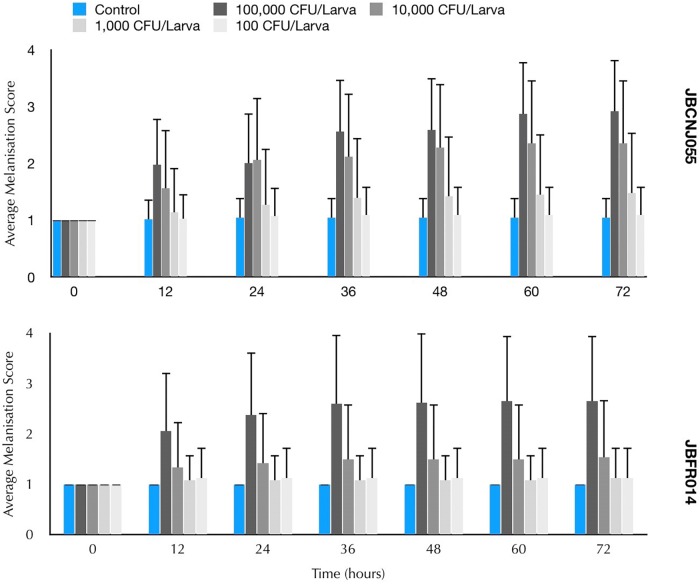
Mean melanisation score of pooled data, 72 h post inoculation of 10 μL of a range of dilutions from 10^7^ to 10^3^ CFU/mL of *Clostridium perfringens* JBCNJ055 and JBFR014. Scoring system is as follows: 1 = healthy, 2 = < 50% melanisation, 3 = > 50% melanisation, 4 = dead. Data shown is pooled from three distinct repeats and error bars represent standard deviation. Injection with 10 μl of 10^7^ was required to cause potent melanisation resulting in scores higher than 3. Survival percentages of *G. mellonella* after inoculation with 10 μL of a range of dilutions 10^5^–10 CFU/mL of *C. perfringens*.

**FIGURE 5:**
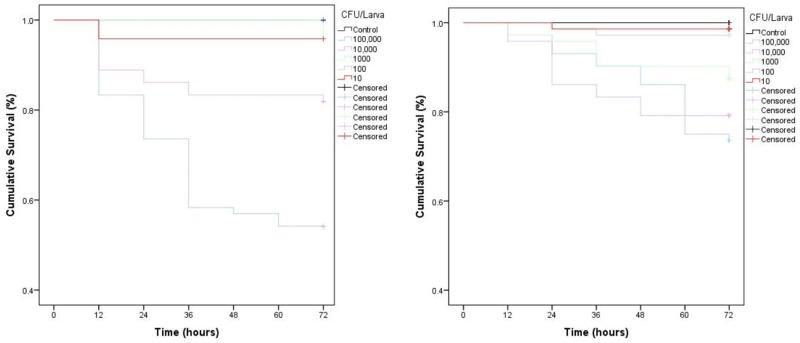
Kaplan–Meier survival distributions for dose dependent challenges of JBCNJ055 and JBFR014. ATCC 13124 is avirulent at all dosage levels. Three repeats of each experiment were pooled. Results were collated as % survival. Strikethrough indicates censored data (unaffected larvae). All levels of infection data are significant (*P* < 0.001) [Mantel (cox) log rank test] for both isolates investigated indicating larval survival is based upon the number of bacteria injected.

Parenteral injection of 10^5^ CFU *C. perfringens* JBCNJ055 resulted in disease of the larvae. Larvae which succumb to the infection exhibited nodulation, blackening of the cuticle, and eventually death. Figure 4 shows the rate of development of infection appears dependent on inoculum size as melanisation score increases rapidly with increasing inoculum doses of *C. perfringens* JBCNJ055. Kaplan–Meier survival distributions for each bacterial inoculum were significant when compared using the log-rank (Mantel-Cox) test (pooled over strata) (*P* < 0.001). Survival probability appears dependent on the number of organisms injected (Figure 5). An inoculum size of 10^5^ CFU/larvae was required for 73.3 ± 6.66% survival and induced melanisation (>3) in >80% of the population. 10^4^ CFU/larva produced 79.2 ± 7.2% survival, however, <30% of the population exhibit melanisation scores higher than 2. Further dilutions (10^3^; 10^2^; 10) produced >90% survival. Injection with 10^5^ CFU/Larva of JBFR014 did incite melanisation in ∼40% of the population, however, survival remained at >90%. Injection with lower dilutions (10^3^; 10^2^; 10) resulted in no visible blackening of the larvae. Interestingly, injection with 10 μL of 10^7^ CFU/mL ATCC 13124 *C. perfringens* did not cause visible disease to the larvae within the 72 h window investigated. Melanisation did occur at the site of injection, however, no further immune response was visually observed. Survival remained above 95% at each inoculum group investigated.

### Phenoloxidase

The results of the phenoloxidase assays shows differences between the control and inoculated groups but not at a significant difference level. Infected groups demonstrated a melanisation score of 3 ± 0.8 at 48 h post inoculation compared to no melanisation in 0.1% peptone controls.

### Cell-Free Toxicity

**FIGURE 6:**
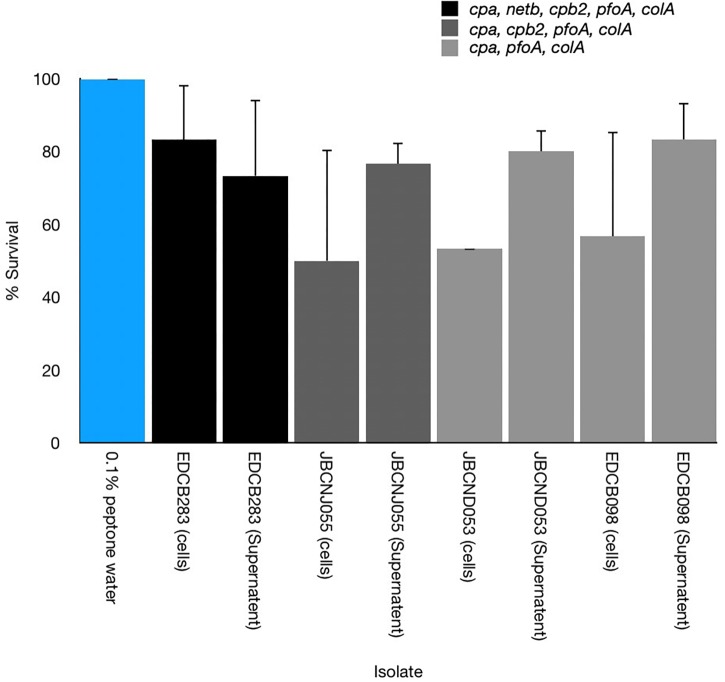
Comparison of the pathogenicity of cell-free supernatants and washed cultures toward *G. mellonella* of three distinct *C. perfringens* isolates with differing virulence gene composition. Cell-free supernatants were able to cause disease but with varying degrees of potency. Error bars represent standard deviation.

All washed harvested cells of *C. perfringens* isolates tested were shown to cause disease in larvae with varying degrees of potency. Furthermore cell-free supernatants of these isolates appeared as potent or demonstrated increased potency compared to uninfected controls of larvae (Figure 6).

### Histopathology of *Galleria* Samples

**FIGURE 7:**
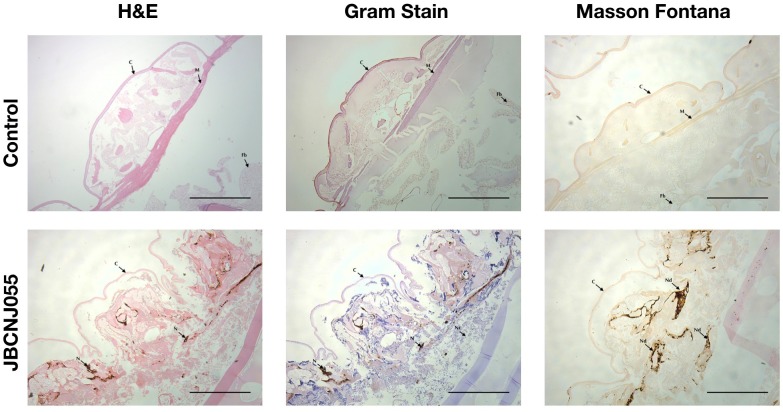
Comparison of *G. mellonella* histopathology of control and infected groups after 72 h incubation after inoculation with 10^5^ CFU of JBCNJ055. Sections were stained with H+E, Gram-stain and Masson Fontana (highlighting melanin deposition). Infected groups show distinct loss of tissue structure, systemic proliferation of Gram-positive bacteria and a large production of melanin within peripheral tissues indicative of systemic pathogenicity. Scale bars represent 1 mm. C, Cuticle; M, Muscle layer; N/Nd, Nodules; Fb, Fat body; Nc, Necrosis.

Figure 7 shows a distinct response of larvae to *C. perfringens* demonstrating aggregation and nodulation of haemocytes compared to uninfected controls There is clear loss of structure and tissue damage in samples which had been injected with 10^5^ CFU/Larva of *C. perfringens* JBCNJ055, with an abundance of melanin demonstrated by Masson Fontana staining compared with negative uninfected controls. Gram-positive cells characteristic of Clostridia were observed associated with tissue remnants in larval sections from *C. perfringens* infected larvae.

### Antibiotic Therapy

**FIGURE 8:**
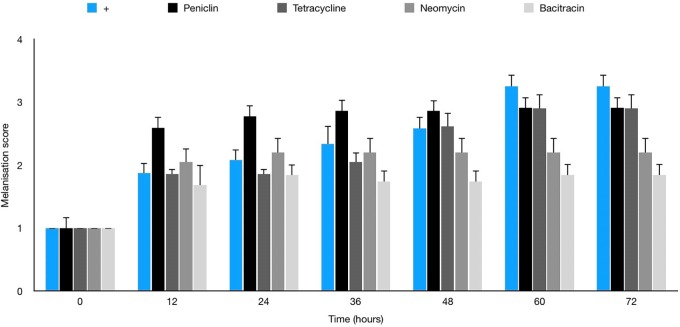
Average melanisation score for larvae infected with 10 μL of 10^7^ JBCNJ055 with a further injection of either penicillin G (2 mg/kg), bacitracin (64 mg/kg), tetracycline (64 mg/kg), or neomycin (2400 mg/kg). Healthy larvae with no signs of infection were scored 1. Larvae showing nodulation and lateral line melanisation scored 2. Larvae exhibiting systemic melanisation scored 3. Dead, fully pigmented larvae scored 4. There is a potent increase in melanisation score when penicillin G is used in response to the infection.

No melanisation or death of larvae was observed with any antibiotics in the absence of infection prior to therapy trials (data not shown). Table 3 shows penicillin G to be the most effective agent *in vitro* against JBCNJ055 (MIC: 0.18 μg/mL), however, *in vivo* larval survival was reduced by 7.5% compared with controls. Interestingly, Figure 8 shows that the penicillin G treatment in infected larvae resulted in a rapid onset of melanisation compared with controls. We have observed a potent systemic melanisation in >80% of the larvae in under 60 min after the treatment dose was administered. Injection of penicillin G alone was not sufficient to cause this potent reaction. Both tetracycline and bacitracin were effective against *C. perfringens* JBCNJ055 *in vitro* (MIC: 1.56 μg/mL). *In vivo*, tetracycline increased larval survival by 7.5% whereas bacitracin greatly increased larval survival by a remarkable 28.3% making it the most effective agent *in vivo* (Table 3). Unsurprisingly, neomycin was the least effective *in vitro* (MIC: 60 μg/mL) alongside a reduction in larval survival *in vivo* of 11.3%.

## Discussion

*Galleria mellonella* is becoming a well established *in vivo* model for virulence studies of a number of important microbes and is featuring prominently in the literature as more pathogens are investigated. The advantages of an insect model over mammalian studies in terms of ethics, speed and research cost are becoming apparent with increasing data (Jander et al., 2000). The time lapse features developed here allows for reduced labor inputs although it is not possible to physically manipulate the larvae, a common practice in assessing larval mortality (Peleg et al., 2009; Harding et al., 2013; Beeton et al., 2015). This has been overcome in the present study by classifying dead insects by a melanisation score of 4 (Figure 1) coupled with no movement for 20 min (2 frames). Time-lapse methodologies, however, increase precision by reducing intermittent 12 h observations to 10 min increments. The increase in precision is of benefit when studying pathogens with rapid disease progression. Inherent bias in the manual scoring of the video should be noted but this can be reduced by our current development of an automated melanisation recognition system utilizing the time-lapse feature which will allow for a more robust and standardized scoring system and reduced processing time.

### *Galleria mellonella* and *Clostridium perfringens*

*Clostridium perfringens* is a ubiquitous organism, with a potential arsenal of toxins and hydrolytic enzymes associated with pathogenicity (Petit et al., 1999). The anaerobic pathogen is economically devastating to the global food industry and is estimated to cost the poultry sector alone over $6 billion (USD) per year (Wade et al., 2015). We have shown for the first time *C. perfringens* is pathogenic toward *G. mellonella* larvae although the degree of pathogenicity is distinct between isolates. The differences in pathogenicity of isolates may be due to the differing toxin expression or environmental factors which are thought to play a major role in mammalian CPAD (Borda-Molina et al., 2018). Interestingly we have shown that cell-free supernatants are able to induce disease indicating that soluble factors (toxins) have pathogenic potential compared with negative controls. *C. perfringens* were able to produce larval death at culture inocula equivalent to other bacteria that have been studied such as *C. jejuni* (10^6^ CFU/Larva) *Y. pseudotuberculosis* (10^6^ CFU/Larva) and *C. difficile* (10^5^ CFU/per larva) (Senior et al., 2011; Champion et al., 2009; Nale et al., 2016), respectively. *C. difficile* is currently the only clostridial species which has been investigated in detail although *C. difficle* virulence data in the context of the *G. mellonella* model is limited. An inoculum size of 10^5^ CFU of *C. difficile isolate CD105LC2* was sufficient to cause 100% mortality in larvae (Nale et al., 2016). The variance in lethality between isolates can be seen in other anaerobic enteric pathogens, however, such as *campylobacter* spp. (Senior et al., 2011), *Listeria* spp. (Martinez et al., 2017), and some strains of enteric *Escherichia* spp. (Jønsson et al., 2017). Essential virulence factors in alternative anaerobes such as *Listeria monocytogenes* have been identified such as the extracellular cytolysin, “listerolysin O” that is essential for virulence, bacterial growth and colonisation of larvae (Joyce and Gahan, 2010).

CPADs are complex and multifactorial processes are still poorly understood (Silva and Lobato, 2015). The use of larval models offers a viable alternative approach for high throughput screening of potentially pathogenic genotypes of anaerobes. In the context of *C. perfringen*s, the model would greatly benefit from further validation studies i.e., the investigation of immunological aspects of infection such as haemocyte viability (Bergin et al., 2005), direct toxicological studies and a variety of histopathological approaches (Harding et al., 2013) to further our understanding of pathogenic processes. Investigation of oral inoculation of *C. perfringens* in *G. mellonella* remains unexplored but would offer data on enteric focused pathogenesis and effects on the microbiome (Mukherjee et al., 2013) that is more analogous to that of mammalian clostridial enteritis and therefore a natural further step in the research.

In the present study, *C. perfringens* activates the phenoloxidase pathway although our enzyme activity data suggests no upregulation of the enzyme in response as seen with other pathogens (Harding et al., 2013). The larvae may allow for an *in vivo* approach to the further understanding of virulence factors that is challenging in mammals. The potential strength of the model lies with the ability to demonstrate the *in vitro* virulence diversity. Broader studies are required to draw conclusions about to what extent the mechanism(s) of pathogenicity in larvae relate to mammalian infections.

In our present study, treatment of the *C. perfringens* infection model with antimicrobials appears to broadly correlate with the *in vitro* data obtained (Table 3). Penicillin G, interestingly produces a robust and potent reaction by the larvae when administered to *C. perfringens* infected larvae which was not seen when the antibiotic was administered to non-infected larvae. Previous studies (Büyükgüzel and Kalender, 2007) have highlighted that penicillin G induces high levels of oxidative stress in the midgut of *G. mellonella* which may predispose the larvae to disease and may explain the inefficiency of penicillin in the model which is well known to be highly effective against *C. perfringens*. Tetracycline and bacitracin are both equally effective *in vitro* against JBCNJ055 but the efficacy of bacitracin *in vivo* is likely due to its more bactericidal nature against Gram-positive organisms in comparison to the broad spectrum but bacteriostatic tetracycline. The introduction of antifungal compounds to *G. mellonella* apparently induces independent immune responses potentially masking the efficacy of drugs (Kelly and Kavanagh, 2011). Further research is required here to ensure that antibacterial drug treatments demonstrate no compounding effects on larval immunity. Hence, the model is potentially useful to investigate novel and experimental treatment approaches in the presence of innate immunological factors without the expense or ethical implications of working with mammals. The data obtained may be used to inform and reduce the number of mammals used for selecting potential lead compounds.

Our methodological advancements described here will improve the ability of the model to evaluate the virulence of pathogens and therapeutic compounds in a more convenient and less laborious way. The data shown in this study suggest that virulence of *C. perfringens* in larvae is distinct and that future studies to dissect the individual contribution of toxin genes in virulence are in hand. We consider that the larvae model will certainly not replace mammalian trials in physiological studies of disease or mechanisms of host response but will be a useful adjunct to potentially characterize unknown pathogenic virulence factors for anaerobic bacteria and perhaps allow pre-screening of antimicrobials against pathogens before embarking on expensive and demanding mammalian studies.

## Author Contributions

RD designed, initiated and overall supervision of project, and critique of the manuscript. JB supported the design of the project and day to day supervision of the laboratory, and supported the preparation of the manuscript. JE undertook partial experimentation and developed the methodology. SK undertook the majority of the experimentation and initial preparation of manuscript.

## Conflict of Interest Statement

The authors declare that the research was conducted in the absence of any commercial or financial relationships that could be construed as a potential conflict of interest.
